# Control of the Thermoelectric Properties of Mg_2_Sn Single Crystals via Point-Defect Engineering

**DOI:** 10.1038/s41598-020-58998-1

**Published:** 2020-02-06

**Authors:** Wataru Saito, Kei Hayashi, Jinfeng Dong, Jing-Feng Li, Yuzuru Miyazaki

**Affiliations:** 1 Department of Applied Physics, Graduate School of Engineering, Tohoku University, Sendai, 980-8579 Japan; 2 State Key Laboratory of New Ceramics and Fine Processing, School of Materials Science and Engineering, Tsinghua University, Beijing, 100084 China

**Keywords:** Electronic properties and materials, Thermoelectrics

## Abstract

Mg_2_Sn is a potential thermoelectric (TE) material that can directly convert waste heat into electricity. In this study, Mg_2_Sn single-crystal ingots are prepared by melting under an Ar atmosphere. The prepared ingots contain Mg vacancies (V_Mg_) as point defects, which results in the formation of two regions: an Mg_2_Sn single-crystal region without V_Mg_ (denoted as the single-crystal region) and a region containing V_Mg_ (denoted as the V_Mg_ region). The V_Mg_ region is embedded in the matrix of the single-crystal region. The interface between the V_Mg_ region and the single-crystal region is semi-coherent, which does not prevent electron carrier conduction but does increase phonon scattering. Furthermore, electron carrier concentration depends on the fraction of V_Mg_, reflecting the acceptor characteristics of V_Mg_. The maximum figure of merit *zT*_max_ of 1.4(1) × 10^−2^ is realised for the Mg_2_Sn single-crystal ingot by introducing V_Mg_. These results demonstrate that the TE properties of Mg_2_Sn can be optimised via point-defect engineering.

## Introduction

Thermoelectric (TE) materials, which are capable of converting waste heat into electricity, are expected to play a significant role in future energy utilisation and management^1,2^. The performance of TE materials is evaluated in terms of the dimensionless figure of merit, *zT* = *S*^2^*σT*/*κ*, and power factor PF = *S*^2^*σ*, where *S*, *σ*, *T* and *κ* are the Seebeck coefficient, electrical conductivity, absolute temperature and thermal conductivity, respectively. Recently, point defects (interstitials, vacancies and antisite defects) in TE materials have been recognised as an important factor that significantly affects the TE performance in the following two ways. First, the carrier concentration in a TE material can be optimised by tuning the fraction of point defects because they can act as acceptors or donors, which results in an increase in PF. Second, thermal conductivity can be reduced by introducing point defects because they can act as phonon scattering centres. Although enhanced *zT* values have been realised for a variety of TE materials via point-defect engineering^3–15^, the fraction of point defects has rarely been evaluated quantitatively. Thus, point-defect engineering cannot yet be considered an established strategy to enhance TE performance.

Recently, our group performed the crystal structure analysis of polycrystalline Mg_2_Si samples and quantitatively verified the presence of interstitial defects in the samples using single-crystal X-ray diffraction (SC-XRD)^16,17^. In the present study, we focus on Mg_2_Sn with the same crystal structure as Mg_2_Si. Mg_2_Sn has been studied extensively as a potential TE material^18–37^. Xin *et al*.^31^ reported that an Sb-doped n-type polycrystalline Mg_2_Sn, Mg_2.15_Sn_0.98_Sb_0.02_ exhibited a *zT* value of 0.53 at 650 K (*κ* and PF are 4.7 W/mK and 3.9 W/mK^2^, respectively). Chen *et al*.^22^ reported that an Ag-doped p-type polycrystalline Mg_2_Sn, Mg_2_Sn + 0.5 at. % Ag exhibited a *zT* value of 0.30 at 450 K (*κ* and PF are 4.0 W/mK and 2.6 W/mK^2^, respectively). Despite these sufficiently high PF values, the *κ* values are also high, limiting the *zT* values lower than ~0.5. Although the importance of controlling point defects in Mg_2_Sn is highlighted in some literature^29,31^, quantitative analysis of point defects has not been performed so far.

Regarding the TE properties of pristine Mg_2_Sn, single crystals exhibited different *S* values depending on the preparation method, as shown in Table 1^18–23^. Generally, different *S* values reflect differences in carrier concentration. Liu *et al*.^32^ predicted that possible point defects in Mg_2_Sn are Mg vacancies (V_Mg_) at the 8*c* (1/4 1/4 1/4) site and Mg interstitial defects (Mg_i_) at the 4*b* (1/2 1/2 1/2) site. These point defects are ionised and generate hole and electron carriers in Mg_2_Sn, respectively, i.e. V_Mg_ → V_Mg_^2−^ + 2 h^+^ and Mg_i_ → Mg_i_^2+^  + 2e^−^. Thus, it is suggested that the difference in *S* values between Mg_2_Sn single crystals prepared using different methods arises from varying fractions of V_Mg_ and/or Mg_i_. Li *et al*.^23^ reported that Mg_2_Sn single crystals exhibited anisotropic *S* values. However, it is possible that the fractions of V_Mg_ and/or Mg_i_ in the single crystals used were different because each Mg_2_Sn single crystal along the [111], [1$\bar{1}$0], or [11$\bar{2}$] direction was prepared by cutting from different parts of one Mg_2_Sn single-crystal ingot.

**Table 1:** Reported *S* and *σ* values for Mg_2_Sn measured at 300 K.

Preparation conditions				Crystalline	*S*_@300 K_ (μV/K)	*σ*_@300 K_ (S/cm)	Ref.
Method	Composition	Melting temp. (K)	*P*_Ar_ (atm) ^c^				
Melt growth	Mg_2_Sn	1093	0.8	PC^d^	−45	23	^18^
	Mg_2.04_Sn				−66	1350	
	Mg_2.05_Sn	1073	0.8		−53	22	^19^
	Mg_2.06_Sn				−39	34	
	Mg_2+*δ*_ Sn	1123	10^−10^		−23	25	^20^
Bridgeman	Mg_2.1_Sn	1093	0.8	SC^e^	−52	26	^21^
RF^a^	Mg_2.1_Sn	1093	0.8	PC^d^	50	27	^22^
HGDS^b^	Mg_2.34_Sn	1200	0.03	SC^e^	−261 (parallel to [111])	49	^23^
					159 (parallel to [1–10])	29	
					149 (parallel to [11–2])	30	

^a^Radio Frequency (RF).

^b^High-temperature Gradient Directional Solidification (HGDS).

^c^Ar pressure (*P*_Ar_).

^d^Polycrystal (PC).

^e^Single-crystal (SC).

To reveal the relationship between point defects and the TE properties of Mg_2_Sn, we prepared Mg_2_Sn single-crystal ingots by melting under an Ar atmosphere. In the prepared ingots, V_Mg_ exists as a point defect, the fraction of which was successfully evaluated by SC-XRD. Furthermore, the V_Mg_ fraction is controlled by changing the Ar pressure, *P*_Ar_. We find a peculiar nanostructure relating to V_Mg_ via transmission electron microscope (TEM); the Mg_2_Sn single crystals with V_Mg_ form nanometer-sized regions and are embedded in the Mg_2_Sn single-crystal region without V_Mg_. The TE properties (|*S*|, *σ* and *κ*) of the ingots all decrease with increasing V_Mg_ because of a decrease in the electron carrier concentration and an enhancement of phonon scattering. In particular, the *κ* value is extremely low compared with that of polycrystalline Mg_2_Sn reported in the literature^28^, which is discussed in relation to the nanostructure of the ingots.

## Results and Discussion

### Phase characterisation and crystal structure refinement

**Figure 1:**
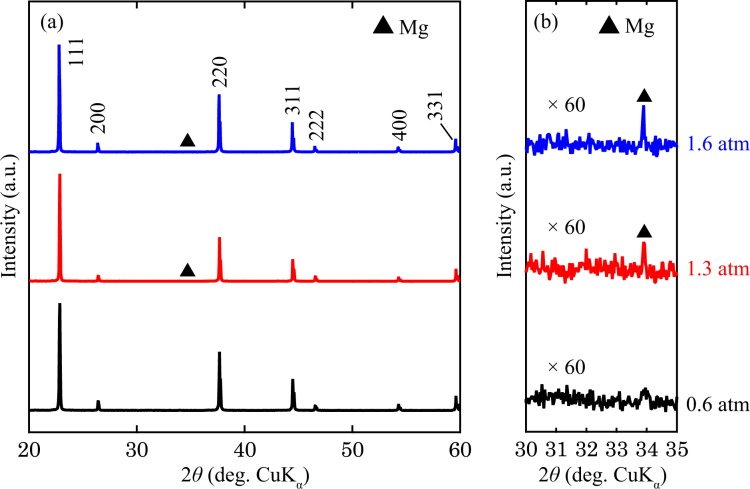
(**a**) Powder X-ray diffraction patterns of the prepared Mg_2_Sn ingots and (**b**) an enlargement of the regions between 30° and 35°.

Figure 1 shows the powder XRD patterns of the Mg_2_Sn single-crystal ingots prepared under *P*_Ar_ = 0.6, 1.3 and 1.6 atm (hereafter, these ingots are referred to as the 0.6-, 1.3- and 1.6-atm ingots, respectively). All of the XRD peaks are well indexed to the expected Mg_2_Sn phase, except for a small peak assigned to an Mg secondary phase found for the 1.3- and 1.6-atm ingots. The bulk XRD measurements confirm the high crystallinity of the ingots. In the bulk XRD patterns, peaks corresponding to the 111, 222 and 333 planes are found (Fig. S1a). In addition, the full width at half maximum of the rocking curves of the 111 peak are as small as 317.3, 438.8 and 453.9 arcsec for the 0.6-, 1.3- and 1.6-atm ingots, respectively (Fig. S1b). A homogeneous distribution of the constituent elements is observed in the SEM-EDX mapping images of the Mg_2_Sn(111) cleavage surface, as shown in Fig. S2, further verifying the high quality of the prepared ingots. The Mg secondary phase is not observed in the SEM-EDX images because of its insignificant amount.

**Figure 2:**
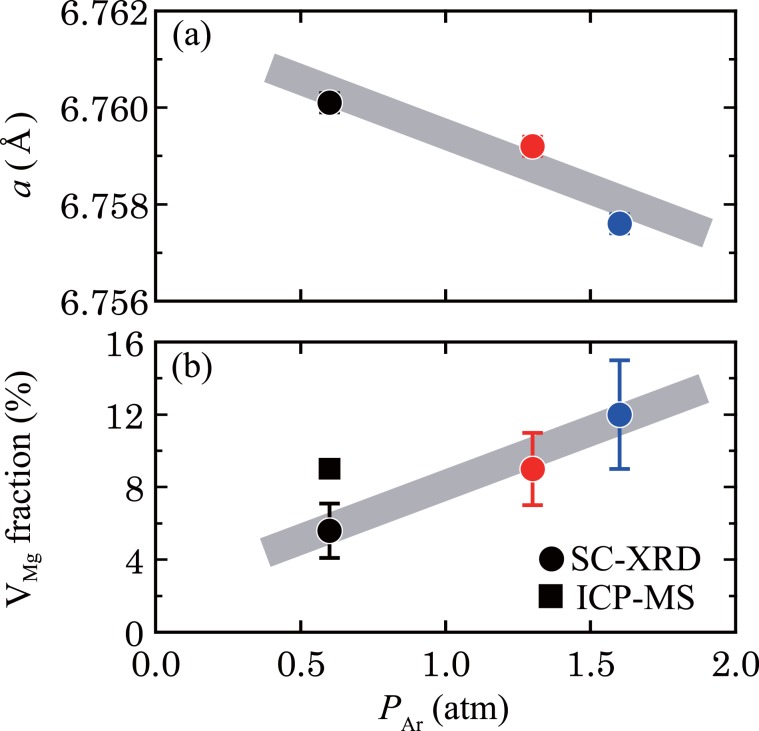
Dependence of the (**a**) lattice constant and (**b**) V_Mg_ fraction evaluated by single-crystal X-ray diffraction (SC-XRD) and inductively coupled plasma mass spectrometry (ICP-MS) on Ar pressure, *P*_Ar_.

Single-crystal structure refinement was performed to evaluate the fraction of point defects (a detailed description can be found in the Supporting Information). As shown in Fig. 2a, the lattice constant decreases from 6.7625(2) Å to 6.7576(2) Å with increasing *P*_Ar_, suggesting a change in chemical composition and/or mutual substitution between Mg and Sn. In other words, the fraction of point defects changes. In fact, the Mg_2_Sn single-crystal ingots contain V_Mg_, the fraction of which increases from 5.6(15)% to 12(3)% with increasing *P*_Ar_ (Fig. 2b). The presence of V_Mg_ is confirmed by the inductively coupled plasma mass spectrometry (ICP-MS) measurement for the 0.6-atm ingot. These results indicate that the V_Mg_ fraction is controllable by changing *P*_Ar_. The increase in V_Mg_ induced by increasing *P*_Ar_ can be attributed to the decreased formation energy of V_Mg_, as reported for the case of Si that formation energy for a Si vacancy is calculated to decrease with increasing hydrostatic pressure^38^.

### Nanostructure characterisation

**Figure 3:**
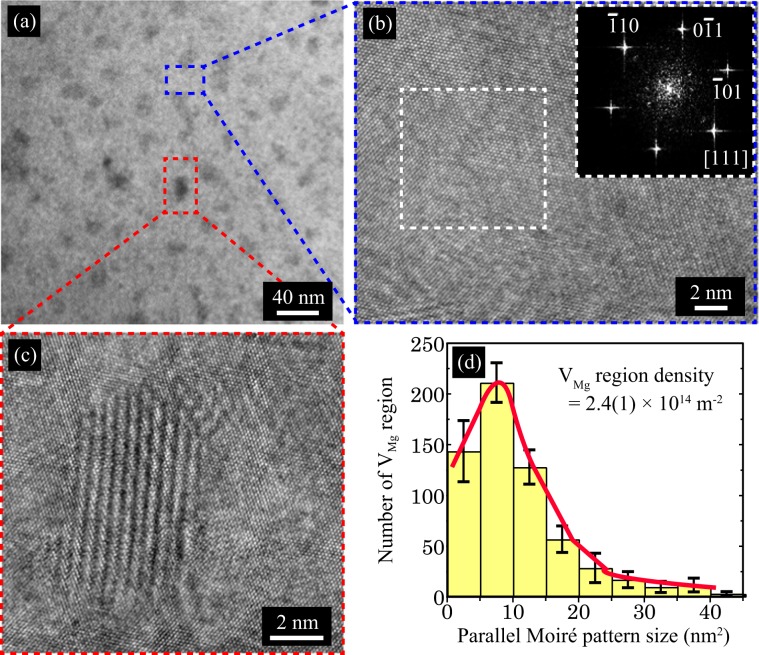
(**a**) Low-magnification transmission electron microscopy (TEM) image of the 0.6-atm ingot. (**b**) High-magnification TEM image of the blue dashed square region in (**a**). The inset shows a fast Fourier transform (FFT) image of the white dashed square region. (**c**) High-magnification TEM image of the red dashed square region in (**a**), in which a parallel Moiré pattern is observed. (**d**) Size distribution of the parallel Moiré pattern corresponding to the location of the Mg_2_Sn single crystal with Mg vacancies (namely, the V_Mg_ region).

The nanostructure of the 0.6-atm ingot was observed using TEM. Figure 3a shows a low-magnification TEM image, in which white and grey regions are observed. The grey regions are dispersed in the white region spread throughout the ingot. As shown in Fig. 3b, a high-magnification TEM image of the white region exhibits a perfect atomic arrangement of the Mg_2_Sn(111) surface, indicating that the white region corresponds to the Mg_2_Sn single-crystal region without V_Mg_ (denoted as the single-crystal region). This is confirmed by the fast Fourier transform (FFT) image of the white region, which shows the diffraction pattern of Mg_2_Sn along the [111] orientation (inset of Fig. 3b).

On the other hand, a high-magnification TEM image of the grey region exhibits a parallel Moiré pattern (Fig. 3c). Generally, a parallel Moiré pattern is observed when the electron beam of the TEM passes through two stacked crystals that have slightly different lattice constants^3,39–43^. Figure S4 shows a simple illustration of this situation. In the present study, the introduction of V_Mg_ into Mg_2_Sn reduces the lattice constant, as mentioned above (see Fig. 2a). Thus, it is conceivable that the observed parallel Moiré pattern originates from the stacking of the Mg_2_Sn single crystal with V_Mg_ on the single-crystal region. In other words, the Mg_2_Sn single crystals with V_Mg_ form nanometer-sized regions (denoted as V_Mg_ regions) that are distributed in the matrix of the single-crystal region. This means that V_Mg_ tends to aggregate in the ingot, which is considered to be energetically stable because the formation energy of point defects is generally lowered by clustering. The size distribution of the V_Mg_ regions was analysed, and it is plotted in Fig. 3d. The average size and density of the V_Mg_ region are 11 nm and 2.4(1) × 10^14^ m^−2^, respectively.

**Figure 4:**
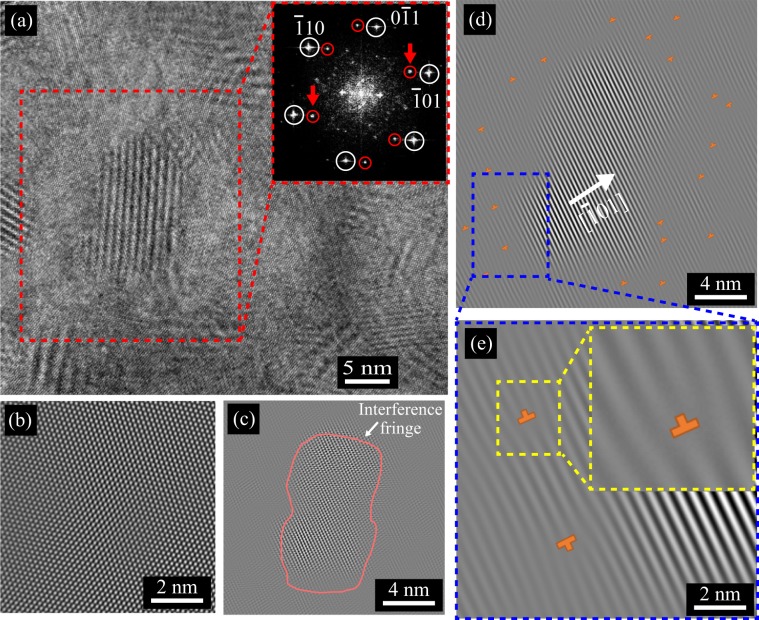
(**a**) High-magnification TEM image of the 0.6-atm ingot. The inset shows a FFT image of the red dashed square region, including the Mg_2_Sn single-crystal regions with and without V_Mg_. Using the diffraction spots marked with the white circles and red circles in the inset, inverse FFT (IFFT) images were generated, as shown in (**b**,**c**), respectively. (**d**) IFFT image transformed from the two spots indicated by red arrows in the inset of (**a**). (**e**) Magnified image of the blue dashed region in (**d**). Dislocations are marked in orange in (**d**,**e**).

To further investigate the interface between the V_Mg_ region and the single-crystal region, an FFT image was produced from the area enclosed in the red dashed square in Fig. 4a, which covers both regions in the 0.6-atm ingot, as shown in the inset of Fig. 4a. The diffraction pattern of the single-crystal region (indicated by the white circles) and some extra spots (indicated by the red circles) are observed. Figure 4b shows the inverse FFT (IFFT) image generated from the diffraction pattern of the single-crystal region, which reproduces the regular atomic arrangement of the Mg_2_Sn(111) surface and is identical to that shown in Fig. 3b. In the IFFT image transformed from the extra spots, some aligned interference fringes are observed around the interface (the red solid line in Fig. 4c). As shown in the filtered IFFT image (Fig. 4d,e) obtained from the two spots in the inset of Fig. 4a (indicated by the red arrows), dislocations (red symbols) can be seen near the interference fringes. From the TEM observation of the 0.6-atm ingot, the dislocation density was evaluated to be 3.5 × 10^16^ m^−2^ in the ingot, indicating that the interface between the V_Mg_ region and the single-crystal region is semi-coherent. Such semi-coherent interfaces are often formed when the lattice mismatch between a precipitate and a matrix is sufficiently small^3,39–43^. The difference in the lattice constants between the Mg_2_Sn single crystals with and without V_Mg_ (corresponding to the V_Mg_ region and the single-crystal region) is smaller than 0.01 Å, as can be seen in Fig. 2a, which can be an origin of the formation of the semi-coherent interface.

### Thermoelectric properties measurements

**Figure 5:**
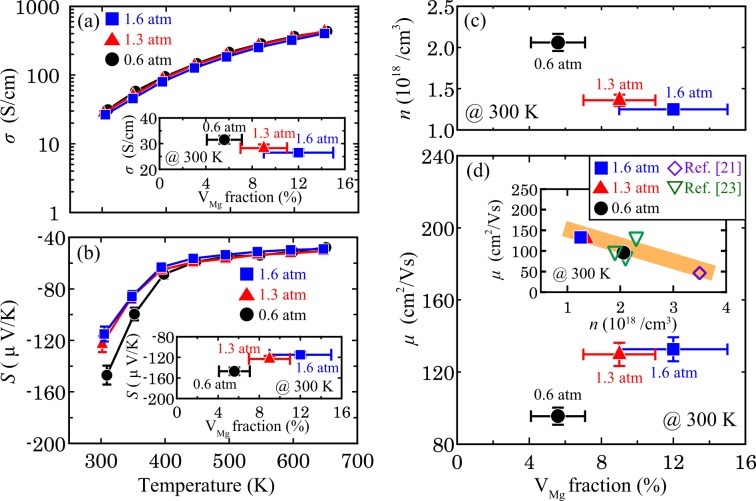
Temperature dependence of (**a**) *σ* and (**b**) *S* of the prepared ingots. These insets show the relation between *σ* and *S* at 300 K and V_Mg_ amount, respectively. Dependence of (**c**) *n* and (**d**) *μ* on the V_Mg_ amount. The inset of (**d**) plots *n* vs. *μ*.

In addition to changing the carrier concentration, the introduction of V_Mg_ may cause carrier and phonon scattering through the formation of nanosized V_Mg_ regions and semi-coherent interfaces. To reveal the relationship between the V_Mg_ fraction and the electrical properties of the Mg_2_Sn, the Seebeck coefficient, *S*, and the electrical conductivity, *σ*, of the 0.6-, 1.3- and 1.6-atm ingots were measured. The temperature dependence of *S* is shown in Fig. 5a. All ingots exhibit negative *S*, i.e. the majority carriers are electrons, which is also confirmed by the negative Hall coefficient of the ingots measured at 300 K. The |*S*| value at 300 K decreases from |−166| μV/K to |−115| μV/K as *P*_Ar_ increases, indicating that the electron carrier concentration is different for each ingot. Upon the increase of the temperature, the differences between the |*S*| values diminish and become negligible above 450 K. These tendencies are attributed to the enhanced bipolar diffusion because Mg_2_Sn is narrow band gap semiconductor^21,23,44,45^. Figure 5b shows the temperature dependence of *σ*. The ingots exhibit similar *σ* values over the entire measurement temperature range. The *σ* value at 300 K slightly decreases from 32 S/cm to 26 S/cm as *P*_Ar_ increases.

The *S* and *σ* values at 300 K are plotted against the V_Mg_ fraction in the insets of Fig. 5a,b, respectively. To determine the reason for the decrease in both |*S*| and *σ* with increasing V_Mg_ fraction, the electron carrier concentration, *n*, at 300 K was evaluated from Hall effect measurements. Figure 5c shows that *n* decreases from 2.23(3) × 10^18^ cm^−3^ to 1.25(1) × 10^18^ cm^−3^ with increasing V_Mg_ fraction, indicating that V_Mg_ acts as an acceptor and cancels a part of the electron carriers in Mg_2_Sn. The change rate of per percentage unit of V_Mg_ is 1 × 10^17^ cm^−3^%^−1^. Generally, |*S*| should increase with decreasing *n*; however, |*S*| decreases in the present study. The controversial behaviour can be explained by assuming that the Fermi level is located near the middle of the band gap. This assumption is reasonable because the ingots are considered intrinsic semiconductors, which is supported by the tendency of *σ* and |*S*| of the ingots to increase and decrease with increasing temperature, respectively, and the fact that the *σ* values at 300 K are as low as several tens of S/cm. In this case, the |*S*| value decreases as the Fermi level moves toward the middle of the band gap upon increasing the V_Mg_ fraction, which is what we observed for the Mg_2_Sn single-crystal ingots in the present study. Turning to the relation between *σ* and *n*, the rate at which *σ* decreases is lower than that for *n*. This means that the carrier mobility, *μ*, increases with increasing V_Mg_ fraction. As shown in Fig. 5d, *μ* increases from 89.9(8) cm^3^/Vs to 132.6(28) cm^3^/Vs, which is inversely proportional to *n*. Such a relation between *n* and *μ* has also been reported for an Mg_2_Si single crystal^46^. The *μ* values of the Mg_2_Sn single-crystal ingots as a function of *n* is shown in the inset in Fig. 5d, which are on an extrapolated line from the reported data of Mg_2_Sn single-crystal ingots^21,23^, implying that V_Mg_ does not act as a carrier scattering centre. From these results, it is concluded that the introduction of V_Mg_ actually changes *n* in the Mg_2_Sn single-crystal ingots, but does not affect *μ*, probably owing to the semi-coherent interface between the V_Mg_ region and the single-crystal region. We should recall that the Mg_2_Sn single-crystal ingots prepared in this study are not uniform in morphology; the V_Mg_ regions are dispersed in the single-crystal region. Thus, the *σ*, *S*, *n* and *μ* values described above are macroscopic physical properties over the whole ingot, i.e., they are actually weighed means considering the contributions from the V_Mg_ regions and the single-crystal region. The changes in the *σ*, *S*, *n* and *μ* values with increasing V_Mg_ fraction originate from the different *σ*, *S*, *n* and *μ* values between the V_Mg_ regions and the single-crystal region. In other words, the V_Mg_ regions have lower *σ*, *S* and *n* values and higher *μ* value than the single-crystal region.

**Figure 6:**
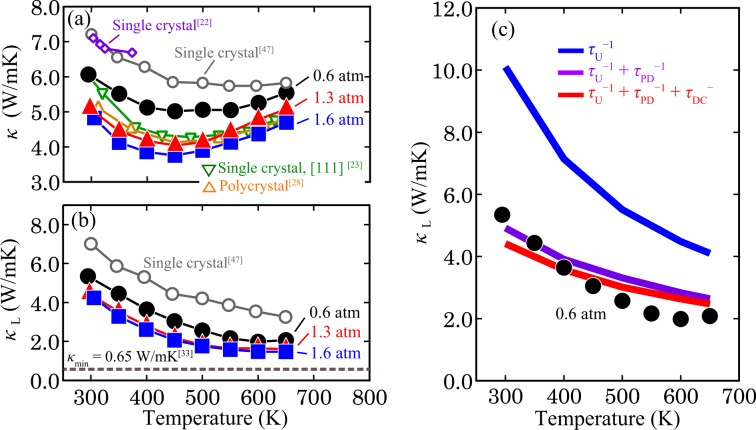
Temperature dependence of (**a**) *κ* and (**b**) *κ*_L_ of the 0.6-atm ingot. (**c**) Comparison of *κ*_L_ with the theoretical calculation. In the calculation, phonon scattering due to the Umklapp process (*τ*_U_^−1^), point defects (*τ*_PD_^−1^) and dislocation cores (*τ*_DC_^−1^) was considered.

Next, the thermal conductivity, *κ*, of the 0.6-, 1.3- and 1.6-atm ingots was measured (Fig. 6a). As *P*_Ar_ increases, i.e. the V_Mg_ fraction increases, *κ* significantly decreases. The 1.6-atm ingot exhibits lower *κ* than the Mg_2_Sn single crystals and polycrystals reported in previous literatures^21,23,25,30^. The minimum *κ* of 3.75(2) W/mK is realised for the 1.6 atm ingot at 450 K. By subtracting the carrier thermal conductivity, *κ*_e_ (Fig. S5a), and the bipolar thermal conductivity, *κ*_bp_ (Fig. S5b), from *κ*, the lattice thermal conductivity, *κ*_L_, was estimated as shown in Fig. 6b. (For the estimation of *κ*_e_, we used the Wiedemann-Frantz law. The deviation of the estimation and the details of the *κ*_e_ and *κ*_bp_ calculations are given in the Supporting Information.) Upon the increase of *P*_Ar_, the *κ*_L_ value of the ingots approaches the minimum calculated *κ*_L_ reported in the literature^31^. The lowest *κ*_L_ of 1.47(1) W/mK is recorded for the 1.6-atm ingot at 650 K, which is still lower than the literature values^21,23,28,47^.

To elucidate the reason for this low *κ*_L_, *κ*_L_ was calculated using the Debye model^48^.

1 $${\kappa }_{L}=(\frac{{k}_{{\bf{B}}}}{2{\pi }^{2}v})(\frac{{k}_{{\bf{B}}}T}{\hslash }){\int }_{0}^{\frac{{\theta }_{{\bf{D}}}}{T}}\,(\frac{{x}^{4}{e}^{x}}{{\tau }_{tot}^{-1}{({e}^{x}-1)}^{2}}dx,\,\,x\equiv \frac{\hslash \omega }{{k}_{{\bf{B}}}T},$$

where $\hslash$, *ω*, *k*_B_, *v*, *θ*_D_ and *τ*_tot_ are the reduced Plank constant, phonon frequency, Boltzmann constant, average sound velocity, Debye temperature and total relaxation time for phonon scattering, respectively. Considering the presence of V_Mg_ and semi-coherent interfaces in the ingot, phonon scattering by point defects and dislocation cores, in addition to the Umklapp process, was included in the calculation. Under these assumptions, *τ*_tot_ is given by *τ*_tot_^−1^ = *τ*_U_^−1^ + *τ*_PD_^−1^ + *τ*_DC_^−1^, where *τ*_U_, *τ*_PD_ and *τ*_DC_ are the relaxation times corresponding to the Umklapp process, point defects and dislocation cores, respectively. More details of the equations expressing these relaxation times and the relevant parameters, such as the average atomic mass, can be found in Tables S4 and S5. As can be seen in Fig. 6c, the calculated result matches well with the experimental *κ*_L_ of the 0.6-atm ingot. It is found that point defects originating from V_Mg_ significantly contribute to the lower *κ*_L_ of the ingot. By contrast, the dislocations at the interface between the V_Mg_ region and the single-crystal region have a smaller effect on *κ*_L_ compared with the point defects probably because the concentration of dislocations (3.5 × 10^16^ m^−2^) is lower than the concentration of point defects (1.3(3) × 10^18^ m^−2^). Therefore, it is concluded that V_Mg_ has the main effect of lowering *κ*_L_ of the Mg_2_Sn single-crystal ingots, but the semi-coherent interface does not lead to as much phonon scattering as V_Mg_.

**Figure 7:**
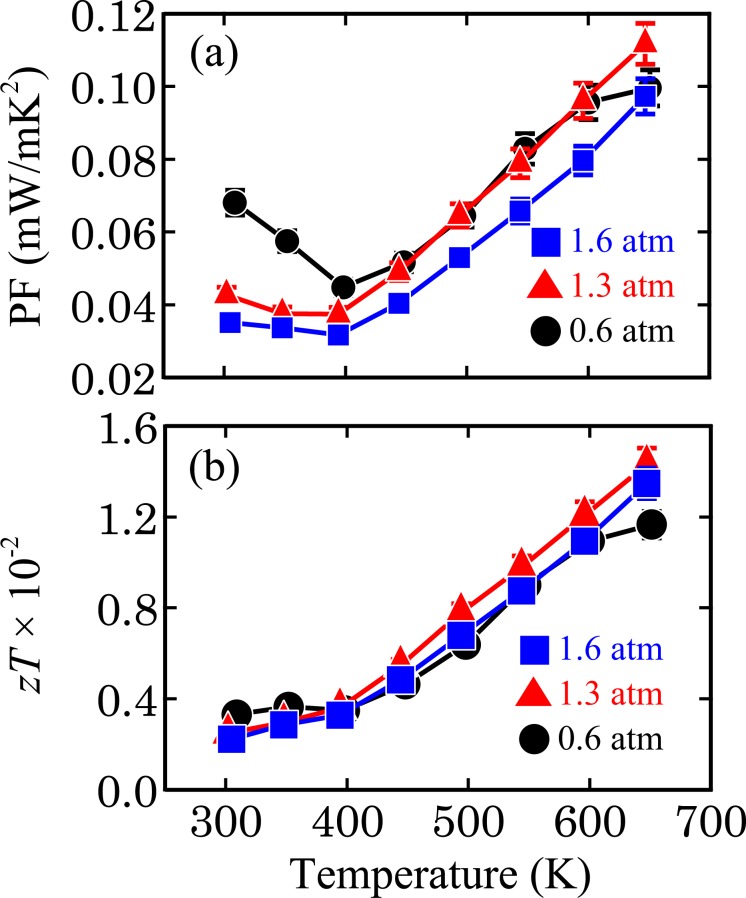
Temperature dependence of (**a**) power factor, PF, and (**b**) dimensionless figure of merit, *zT*, of the prepared ingots.

Finally, the PF and *zT* values of the 0.6-, 1.3- and 1.6-atm ingots were calculated, and the results are plotted in Fig. 7a,b, respectively. At temperatures below 450 K, the PF value decreases with increasing *P*_Ar_, reflecting the variation in |*S*| among the ingots. Above 450 K, the prepared ingots show similar PF values because the difference in *S* and *σ* between the ingots is small, but slightly higher PF values are obtained for the 0.6- and 1.3-atm ingots relative to the 1.6-atm ingot. The highest PF is 0.11(1) mW/mK^2^ at 650 K for the 1.3-atm sample. Regarding *zT*, the 0.6- and 1.3-atm ingots show higher *zT* values than the other ingots in the temperature ranges from 300 to 400 K and from 400 to 650 K, respectively. The maximum *zT* value is 1.4(1) × 10^−2^ at 650 K for the 1.3-atm ingot mainly because of its low *κ*. In this study, we have succeeded in evaluating the V_Mg_ fraction in Mg_2_Sn single-crystal ingots. The TE properties are optimised by controlling the V_Mg_ fraction, demonstrating that point-defect engineering is an effective strategy to enhance the PF and *zT* of Mg_2_Sn. The superiority of the Mg_2_Sn single-crystal ingots with V_Mg_ prepared in this study is low *κ*, but PF and *zT* are still low. (The comparison of *κ*_min_, PF_max_ and *zT*_max_ of the 0.6-, 1.3- and 1.6-atm with those of other Mg_2_Sn single crystals reported in the literatures^21,23^ is shown in Fig. S6.) By combining the reduction of *κ*_L_ (<1.5 W/mK) via point defect engineering, as in this study, with the enhancement of PF (~4.0 mW/mK^2^) via a conventional doping method^31^, a higher *zT* value (*zT* > 0.9 at 650 K) can be achieved for a Mg_2_Sn single crystal.

## Methods

Mg_2_Sn single-crystal ingots were synthesised by melting using a one-zone-controlled resistive-heating furnace. Mg grains (4 N, Mitsuwa Chemicals Co., Ltd, 4 × 4 mm) and Sn powder (4 N, Kojundo Chemical Lab., 63 μm pass) were weighed and charged into a boron-nitride-coated alumina crucible in a molar ratio of Mg:Sn = 2.2:1. The crucible was then enclosed in a quartz tube under an Ar atmosphere of pressure, *P*_Ar_ = 0, 0.6, 1.3, or 1.6 atm at room temperature. The tube was heated to 1093 K over 9 h, then cooled to 973 K slowly over 48 h and finally cooled to room temperature over 9 h.

The crystalline phases and crystallinity of the Mg_2_Sn single-crystal ingots were investigated by powder and bulk X-ray diffraction (XRD) using Cu Kα radiation (D8 ADVANCE, Bruker AXS). For the bulk XRD measurements, the Mg_2_Sn(111) cleavage surface was used. Moreover, a rocking curve of the 111 peak was measured. Owing to the difficulty in crystal structure refinement using the powder XRD patterns of Mg_2_Sn, as was the case for Mg_2_Si^16,17^, the fraction of point defects was evaluated via single-crystal XRD (SC-XRD) using Mo Kα radiation (D8 VENTURE, Bruker AXS). Small Mg_2_Sn single-crystal particles with typical dimensions of 40 μm × 60 μm × 60 μm, selected from fractured Mg_2_Sn single-crystal ingots, were used for the SC-XRD measurements. Single-crystal structure refinement was performed using the JANA2006 crystallographic computing system^49^. The fraction of point defects was also examined by inductively coupled plasma–mass spectrometry (ICP-MS, Agilent 8800 ICP-QQQ, Agilent Technologies). The morphology and nanostructure of the Mg_2_Sn single-crystal ingots were observed using a scanning electron microscope equipped with an energy-dispersive X-ray spectrometer (SEM-EDX; JSM-IT100, JEOL) and a field-emission-type TEM (JEM-2100F, JEOL).

The Seebeck coefficient, *S*, and the electrical conductivity, *σ*, of the Mg_2_Sn single-crystal ingots were measured in vacuum using an automated thermoelectric tester (RZ2001i, Ozawa Science Co.). The Hall coefficient, *R*_H_, was measured using a physical properties measurement system (PPMS, Quantum Design) at 300 K by sweeping the magnetic field from −5.0 T to 5.0 T. The carrier concentration, *n*, and carrier mobility, *μ*, were calculated using the equations *n* = |1/(*eR*_H_)| and *μ* = *σR*_H_, respectively, where *e* is the elementary charge. The thermal conductivity, *κ*, was measured in vacuum using a standard laser flash analyser (TC-7000, ULVAC-RIKO).

## Supplementary information

Supplementary informations.
