# Supplementary material for: Control of the Thermoelectric Properties of Mg2Sn Single Crystals via Point-Defect Engineering
Source: Sci Rep. 2020 Feb 6;10:2020. doi: 10.1038/s41598-020-58998-1 (PMC7005024; doi:10.1038/s41598-020-58998-1)
Supplement: Supplementary file 1 — Supplementary informations. [file 41598_2020_58998_MOESM1_ESM.pdf]

Supporting Information.

Control of the Thermoelectric Properties of Mg_2Sn Single Crystals via Point-Defect Engineering

Wataru Saito,[†] *Kei Hayashi,*[*†] *Jinfeng Dong,*[‡] *Jing-Feng Li,*[‡] *and Yuzuru Miyazaki*[†]

[†] Department of Applied Physics, Graduate School of Engineering, Tohoku University, Sendai 980-8579, Japan

[‡] State Key Laboratory of New Ceramics and Fine Processing, School of Materials Science and Engineering, Tsinghua University, Beijing 100084, China

Corresponding Author: *hayashik@crystal.apph.tohoku.ac.jp

Crystallinity and composition of the Mg_2Sn single-crystal ingots

Figure S1a shows the bulk X-ray diffraction (XRD) measurements of the Mg_2Sn single-crystal ingots prepared under P_{Ar} = 0.6, 1.3 and 1.6 atm (described as the 0.6-, 1.3- and 1.6-atm ingots, respectively). Peaks corresponding to the 111, 222 and 333 planes appear in the XRD patterns of the fractured surface of the ingots, indicating that the ingots are actually single crystals. Figure S1b shows the rocking curves of the 111 peaks in the bulk XRD patterns of the ingots. The full width at half maximum are 317.3, 438.8 and 453.9 arcsec for the 0.6, 1.3 and 1.6 atm samples, respectively. These results confirm the high crystallinity of the ingots. The SEM-EDX mapping images of the Mg_2Sn (111) cleavage surface shown in Figure S2 indicate that the constituent elements are homogeneously distributed, further verifying the high quality of the prepared ingots.

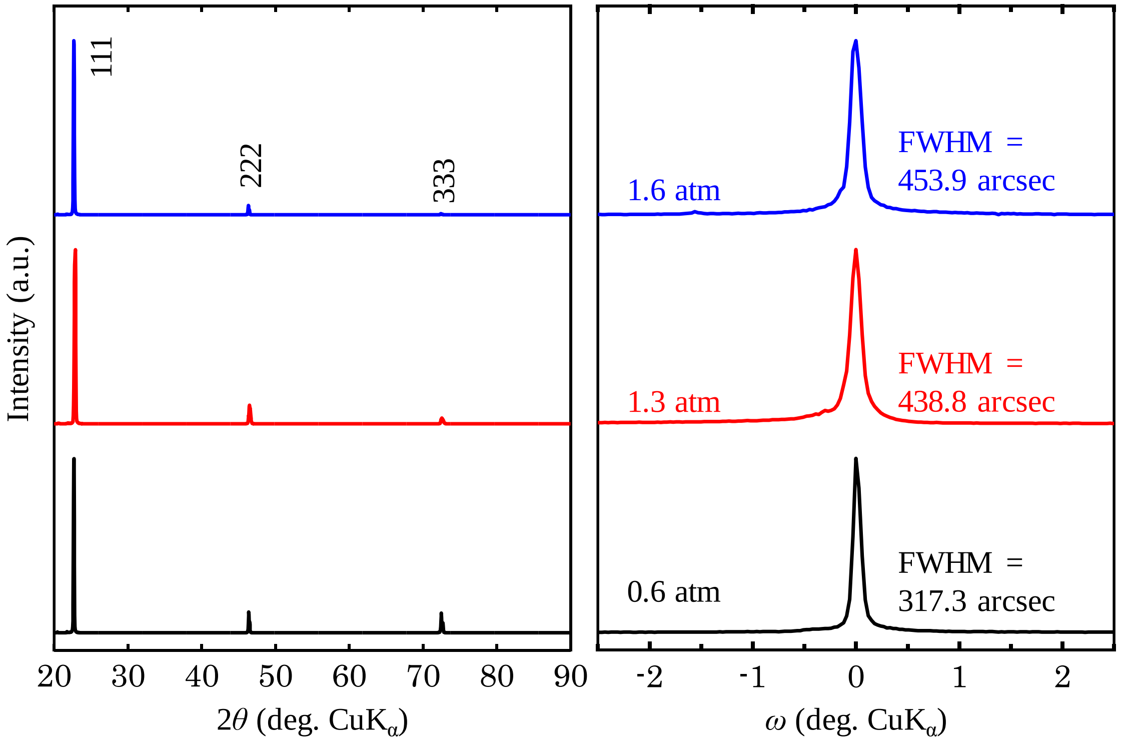

Figure S1. (a) Bulk X-ray diffraction (XRD) patterns of the cleavage surface of the prepared Mg_2Sn ingots and (b) X-ray rocking curves of the 111 XRD peak.

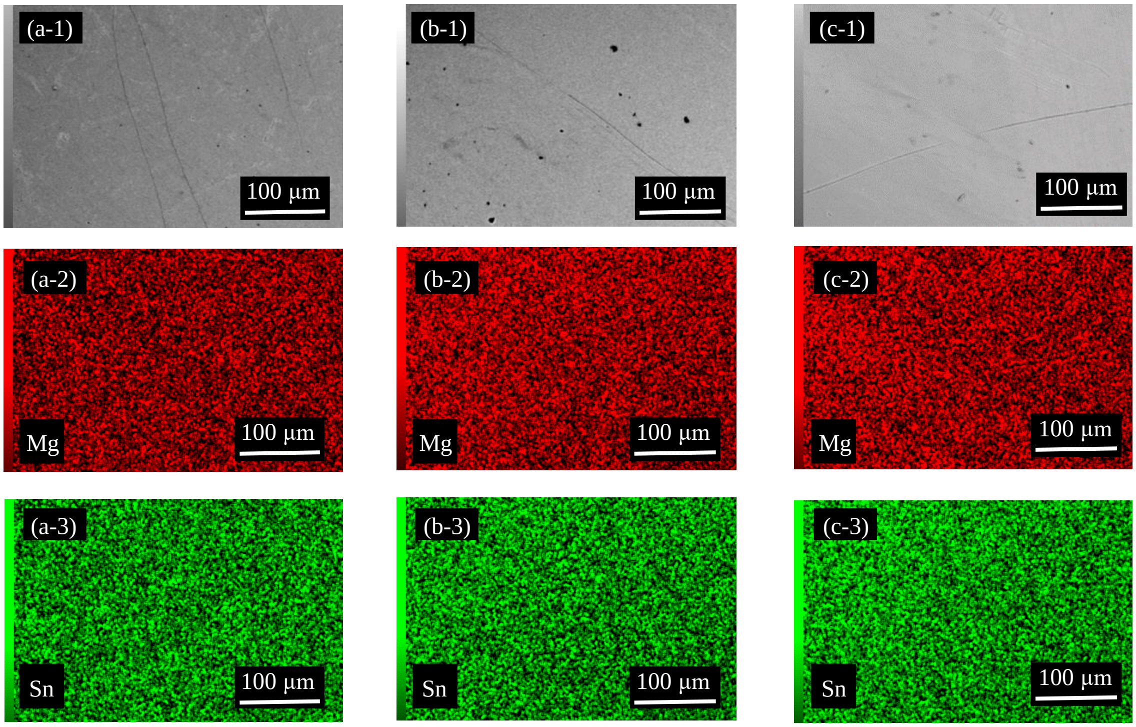

Figure S2. Energy-dispersive X-ray spectroscopy (EDX) mapping of the (a1–3) 0.6-atm ingots, (b1–3) 1.3–atm ingots and (c1–3) 1.6-atm ingots.

Single-crystal structure refinement

For single-crystal structure refinement, three structure models were used: (a) stoichiometric Mg_2Sn, (b) Mg_2Sn with Mg vacancies (V_{Mg}) and (c) Mg_2Sn with V_{Mg} and Mg interstitial defects (Mg_i). In the case of model (c), the Mg_i fraction became negative or zero during structural refinement. Hence, it was concluded that Mg_i does not exist in the ingots. As shown in Table S1, the evaluated *w*R-factor for model (b) is lower than that for model (a). To confirm the significance of the lower *wR*-factor, the Hamilton test [1] was performed. The ratio of *wR-factors*, $R = \frac{R0}{R1}$, where $R0$ and $R1$ are the *wR-factors* for models (a) and (b), respectively, is compared to the reliability factor, $R_{b,n-b,\alpha} = \left[\left(\frac{b}{n-b}\right) F_{b,n-b,\alpha} + 1\right]^{\frac{1}{2}}$, where b, n, α and $F_{b,n-b,\alpha}$ are the increase in the number of model parameters, degrees of freedom, significance level and F-distribution function, respectively. In this study, b is equal to unity because the difference in the number of parameters between model (a) and model (b) is only one parameter, the V_{Mg} fraction and n is the number of unique reflections. Provided that $R > R_{b,n-b,\alpha}$, the hypothesis that V_{Mg} exist in the ingot is not rejected at the significance level, α. Table S1 lists the $R_{b,n-b,\alpha}$ values at the minimum α where $R > R_{b,n-b,\alpha}$. It was found that α was lower than 0.006 for all ingots, indicating that the hypothesis that the crystals contain V_{Mg} should not be rejected at a significance level below 0.006. The significance level of 0.006 is low enough to conclude that the prepared Mg_2Sn single-crystal ingots contain V_{Mg}.

Table S1. Results and significance level obtained by Hamilton test.

sample	R_0	R_1	n	R	$R_{b,\ n-b,\ \alpha}$	α
0.6 atm	1.70	1.63	95	1.0429	1.0428	0.0051
1.3 atm	2.71	2.53	95	1.0711	1.0669	0.0005
1.6 atm	2.03	1.94	95	1.0464	1.0430	0.0050

The existence of V_{Mg} in the Mg_2Sn single-crystal ingots was further examined by using a difference Fourier map. Figure S3a shows the difference Fourier map at the z = 0.25 plane assuming model (a) for the 0.6 atm ingot. The residual electron density is plotted with green (positive) and blue (negative) contour levels. The residual electron density at the 8c (1/4 1/4 1/4) site is negative (−3.15 e/Å^3), indicating the presence of V_{Mg} at this site. Figure S3b shows the difference Fourier map at the z = 0.25 plane assuming model (b). The absolute value of the residual electron density at the 8c site decreases |−0.25 e/Å^3|. Such a change in the electron density is also found for the other ingots. Table S2 lists the refined structural parameters of the prepared ingots. Sn occupancy is 100%, whereas Mg occupancy decreases with increasing P_{Ar}. In other words, the V_{Mg} fraction increases as P_{Ar} increases.

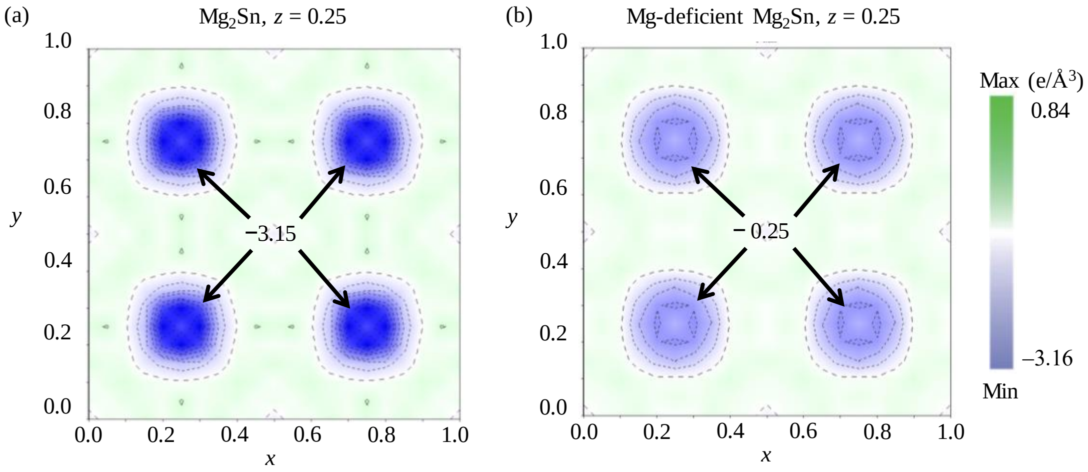

Figure S3. Difference Fourier maps at z = 0.25 plane of the 0.6-atm ingot assuming (a) Mg_2Sn and (b) Mg-deficient Mg_2Sn. The green (blue) areas indicate positive (negative) contour levels.

Table S2. Refined structural parameters of the prepared ingots.

sample	gof	Occupancy of Mg (%)	U_{iso} of Mg (Å^2)	Occupancy of Sn (%)	U_{iso} of Sn (Å^2)
0.6 atm	1.42	94.4 (15)	0.0124 (5)	100	0.00930 (12)
1.3 atm	3.27	91.0 (20)	0.0112 (9)	100	0.00865 (19)
1.6 atm	3.3	88.0 (30)	0.0095 (10)	100	0.00760 (20)

Schematic illustration of transmission electron microscopy

Figure S4 illustrates a simple situation where the electron beam of the transmission electron microscope (TEM) passes through two stacked crystals with slightly different lattice constants. In this situation, a parallel Moiré pattern is observed in the TEM image.

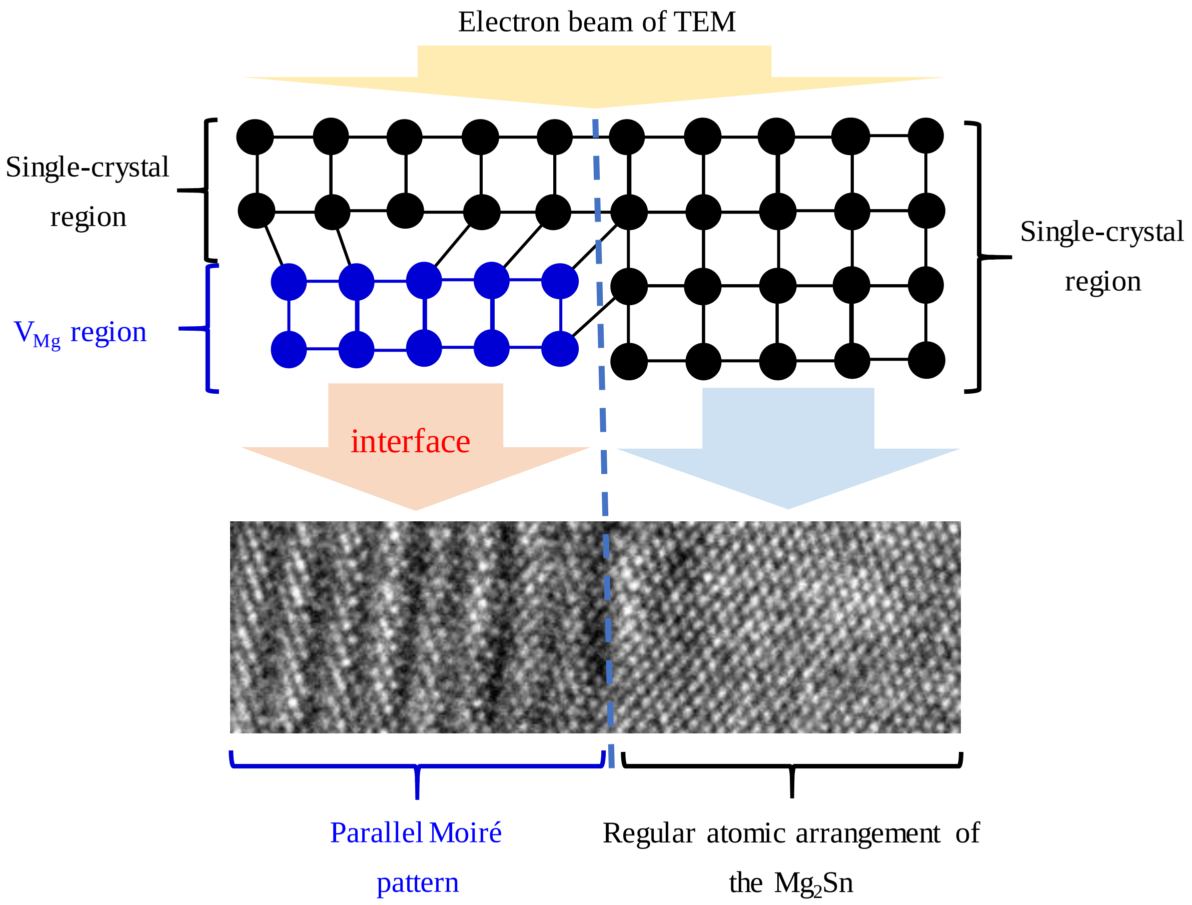

Figure S4. Simple illustration showing that the electron beam of the transmission electron microscope (TEM) passes through two stacked crystals with slightly different lattice constants.

Detailed calculation of the thermal conductivity based on the Debye model

For the evaluation of the carrier thermal conductivity, κ_e, of the ingots, we have to consider the contributions of the V_{Mg} regions and the single-crystal region to κ_e separately, and integrate them according to the generalized effective mean theory [2-4]. Unfortunately, for this evaluation, the number of data points for σ and S is inadequate. Many Mg_2Sn single-crystal ingots with different V_{Mg} fraction must be prepared. The control of the V_{Mg} fraction in a wide range will be performed by changing the preparation temperature and/or the nominal composition in the future. At present, we can only estimate the κ_e, of the ingots using the Wiedemann–Franz law, $\kappa_e = L\sigma T$, where L is the Lorenz number and σ is the measured value of the ingots shown in Fig. 5a. According to the literature [5], L is estimated from the Seebeck coefficient, S:

$$L = 1.5 + \exp\left(-\frac{|S|}{116}\right). \qquad (1)$$

This equation cannot be applied to materials with complex non-parabolic band structures, where L values estimated using eq. (1) deviate from calculated ones by up to 13% [6, 7]. In addition, the eq. (1) is accurate within 5% in the case that acoustic phonon scattering is dominant [5], and for other phonon scattering mechanisms, the deviation reaches as large as 20% [5]. Mg_2Sn is known as an intrinsic semiconductor with a parabolic band structure [8]. However, the dominant phonon scattering mechanism in Mg_2Sn is uncertain; acoustic phonon scattering is adopted in several references [9-12] and optical phonon scattering is considered in another reference [13]. Thus, the deviation is at most 20%. Figure S5a shows the carrier thermal conductivity, κ_e, of the ingots estimated by using eq. (1). In spite of the large deviation, the estimated κ_L of the 0.6 atm ingot by subtracting κ_e and bipolar thermal conductivity, κ_{bp}, from total thermal conductivity is well fitted by a theoretical calculation of κ_L adopting nano-structural features in the 0.6 atm ingot (Fig. 6c). This result ensures the validity of the use of eq. (1).

The κ_{bp} of the ingots is shown in Figure S5b. For the calculation of κ_{bp}, the following equation is used [9]:

$$\kappa_{bp} = \left(\frac{k_B}{e}\right)^2 \left(4 + \frac{E_g - 3\times10^{-4}T}{k_B T}\right)^2 \frac{npb}{bn+p}\sigma T, \qquad (2)$$

where E_g, p and b are the energy band gap, hole concentration, electron mobility, and mobility ratio (= μ_n / μ_p), respectively. These parameters are listed in Table S3. The equations giving the relaxation time (Umklapp process, τ_U [16], point defects, τ_{PD} [17] and dislocation cores, τ_{DC} [18]) and the relevant parameters are listed in Tables S4 and S5, respectively.

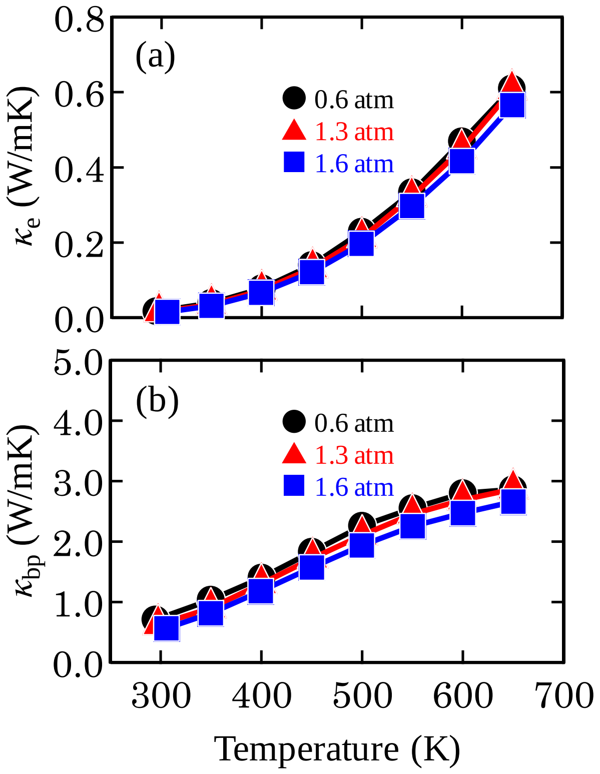

Figure S5. Temperature dependence of (a) κ_e and (b) κ_{bp} of the prepared ingots.

Table S3. Parameters used to calculate κ_{bp}.

Parameter	Value	Ref.
E_g	0.36 eV	[14]
p	$p = n$ (intrinsic semiconductor)	This work
μ_n	89	This work
μ_p	130	[15]

Table S4. Equations for the various phonon relaxation times used in the calculations.

Scattering mechanism	Equation	Ref.
Umklapp processes	$\tau_U^{-1} = \frac{\hbar\gamma^2\omega^2 T}{Mv^2\theta_D} e^{\frac{-\theta_D}{3T}}$	[16]
Point defects	$\tau_{PD}^{-1} = \frac{V\omega^4}{4\pi v^3}\Gamma$	[17]
Dislocation cores	$\tau_{DC}^{-1} = N_{\mathrm{DC}}\frac{V^{\frac{4}{3}}\omega^3}{v^2}$	[18]

Table S5: Parameters used for calculating the relaxation time listed in Table S4.

Parameter	Description	Value	Ref.
γ	Grüneisen parameter	2.1	This work (fitting param.)
M	Average atomic mass	6.9×10^{-26} kg	This work (calc.)
v	Average sound velocity	3000 m/s	[19]
θ_D	Debye temperature	240 K	[20]
Γ	Disorder parameter	55.3×10^{-3} (V_{Mg} fraction : 5%)	[21]
N_{DC}	Dislocation density	3.5×10^{16} m^{-2}	This work (exp.)

Comparison of κ_{min}, PF_{max}, and zT_{max} among the Mg_2Sn single crystals

The κ_{min}, PF_{max} and zT_{max} values of the Mg_2Sn single-crystal ingots prepared in this study and those of the Mg_2Sn single crystals reported in the literatures [13, 22] are shown in Figures S6a, S6b and S6c, respectively. The 1.6 atm ingot prepared in this study exhibited the lowest κ_{min}. In the literatures, the Mg_2Sn single-crystal ingots were prepared under an Ar atmosphere of pressure, P_{Ar} = 0.03 atm [22] and 0.8 atm [13], which are lower than our preparation condition. Thus, it is expected that the ingots prepared in the literatures have less V_{Mg} if any, and the V_{Mg} region is not formed in the ingots. In fact, the actual composition of one of the ingots is reported to be Mg:Sn = 2:1 [22]. Thus, we can attribute the lowest κ_{min} to the introduction of V_{Mg} which forms nano-regions in the single-crystal region.

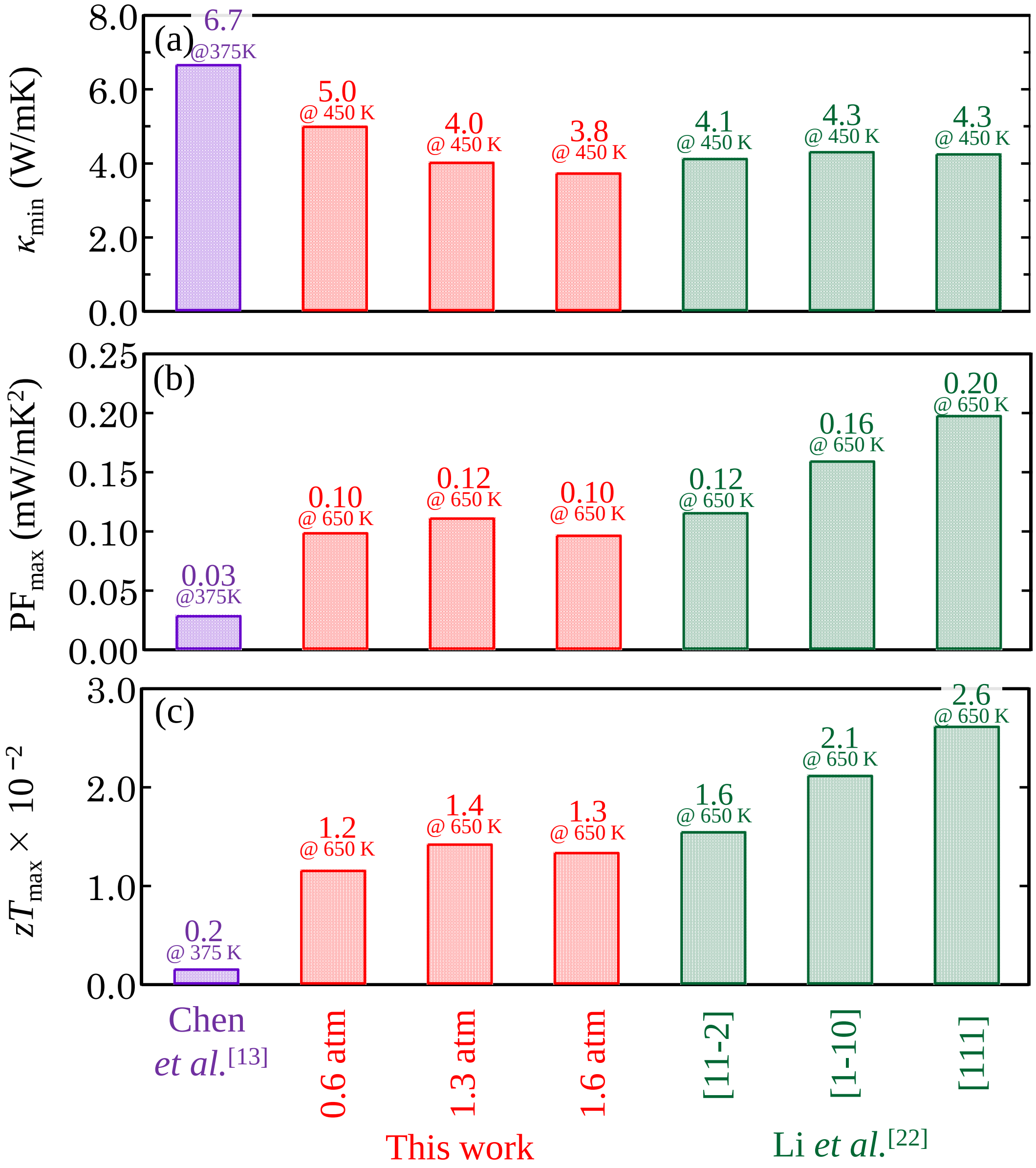

Figure S6. (a) κ_{min}, (b) PF_{max} and (c) zT_{max} of the Mg_2Sn single-crystal ingots prepared in this study. The literature values of Mg_2Sn single crystals [13, 22] are also shown for comparison.

References

(1) Hamilton, W. C. Significance Tests on the Crystallographic R factor. *Acta Crystallogr.* **18**, 502–510 (1965).

(2) MacLachlan, D. S. An equation for the conductivity of binary mixtures with anisotropic grain structures. *J. Phys. C: Solid State Phys.* **20**, 865-877 (1987).

(3) Vaney, J. B. *et al.* Effective medium theory based modeling of the thermoelectric properties of composites: comparison between predictions and experiments in the glass–crystal composite system $Si_{10}As_{15}Te_{75}$–$Bi_{0.4}Sb_{1.6}Te_3$. *J. Mater. Chem. C* **3**, 11090-11098 (2015).

(4) Sonntag, J. Comment on "Effective medium theory based modeling of the thermoelectric properties of composites: comparison between predictions and experiments in the glass–crystal composite system $Si_{10}As_{15}Te_{75}$–$Bi_{0.4}Sb_{1.6}Te_3$" by J.-B. Vaney et al., J. Mater. Chem. C, 2015, 3, 11090. *J. Mater. Chem. C* **4**, 10973-10976 (2016).

(5) Kim, H.-S. *et al.* Characterization of Lorenz Number with Seebeck coefficient Measurement. *APL Mater.* **3**, 041506 (2015).

(6) Berland, K. *et al.* Thermoelectric transport trend in group 4 half-Heusler alloys. *J. Appl. Phys.* **126**, 145102 (2019).

(7) Putatunda, A. and Singh, D. J. Lorenz number in relation to estimates based on the Seebeck coefficient. *Mater. Today Phys.* **8**, 49-55 (2019).

(8) Jin, Y. *et al.* Mg_2Sn: a Potential Mid-temperature Thermoelectric Material. *RSC Adv.* **6**, 48728–48736 (2016).

(9) Martin, J. J. Thermal Conductivity of Mg_2Si, Mg_2Ge and Mg_2Sn. *J. Phys. Chem. Solids* **33**, 1139–1148 (1972).

(10) Kim, S. *et al.* Electronic structures and thermoelectric properties of p-type Ag-doped Mg_2Sn and $Mg_2Sn_{1-x}Si_x$ (x = 0.05, 0.1). *J. Appl. Phys.* **116**, 153706 (2014).

(11) Zhang, L. *et al.* Suppressing the bipolar contribution to the thermoelectric properties of $Mg_2Si_{0.4}Sn_{0.6}$ by Ge substitution. *J. Appl. Phys.* **117**, 155103 (2015).

(12) Mao, J. et al. Thermoelectric performance enhancement of Mg_2Sn based solid solutions by band convergence and phonon scattering *via* Pb and Si/Ge substitution for Sn. *Phys. Chem. Chem. Phys.* **18**, 20726-20737 (2016).

(13) Chen, H. Y. *et al.* Electronic and thermal transport properties of Mg_2Sn crystals containing

finely dispersed eutectic structures. *Phys. Status Solidi A* **207**, 2523-2531 (2010).

(14) Winkler, U. Die elektrischen Eigenschaften der intermetallischen Verbindungen Mg_2Si, Mg_2Ge, Mg_2Sn und Mg_2Pb. *Helv. Phys. Acta.* **28**, 633–666 (1955).

(15) Lipson, H. G. and Kahan, A. Infrared Absorption of Magnesium Stannide. *Phys. Rev.* **133**, A800 (1964).

(16) Slack, G. A. and Galginaitis, S. Thermal Conductivity and Phonon Scattering by Magnetic Impurities in CdTe. *Phys. Rev.* **133**, A253 (1964).

(17) Abeles, B. Lattice Thermal Conductivity of Disordered Semiconductor Alloys at High Temperatures. *Phys. Rev.* **131**, 1906–1911 (1963).

(18) Klemens, P. G. The Scattering of Low-Frequency Lattice Waves by Static Imperfections. *Proc. Phys. Soc.* **A68**, 1113–1128 (1955).

(19) Davis, L. C. *et al.* Elastic Constants and Calculated Lattice Vibration Frequencies of Mg_2Sn. *J. Phys. Chem. Solids* **28**, 439–447 (1967).

(20) Jelinek, F. J. *et al.* Thermal Study of Group II–IV Semiconductors—II. Heat Capacity of Mg_2Sn in the Range 5–300°K. *J. Phys. Chem. Solids* **28**, 267–270 (1967).

(21) Xin, J. *et al.* Multiscale Defects as Strong Phonon Scatters to Enhance Thermoelectric Performance in $Mg_2Sn_{1-x}Sb_x$ Solid Solutions. *Small Methods* 1900412 (2019).

(22) Li, X. *et al.* Anisotropy of Seebeck Coefficient in un-doped Mg_2Sn Single Crystal. *Intermetallics* **81**, 26–31 (2010).
